# Measuring Timeliness of Outbreak Response in the World Health Organization African Region, 2017–2019

**DOI:** 10.3201/eid2611.191766

**Published:** 2020-11

**Authors:** Benido Impouma, Maroussia Roelens, George Sie Williams, Antoine Flahault, Claudia Torres Codeço, Fleury Moussana, Bridget Farham, Esther L. Hamblion, Franck Mboussou, Olivia Keiser

**Affiliations:** World Health Organization Regional Office for Africa, Brazzaville, Congo (B. Impouma, G. Williams, F. Moussana, B. Farham, E.L. Hamblion, F. Mboussou);; Institute of Global Health, University of Geneva, Switzerland (M. Roelens, A. Flahault, O. Keiser);; Fundacao Oswaldo Cruz Ringgold Standard Institution, Rio de Janeiro, Brazil (C.T. Codeço)

**Keywords:** communicable diseases, disease outbreaks, foodborne diseases, Integrated Disease Surveillance and Response strategy, internally-displaced populations, outbreak response, refugee populations, time to control, time to detection, time to notification, vaccine-preventable diseases, vector-borne diseases, waterborne diseases, WHO African Region, WHO Health Emergencies Programme, World Health Organization

## Abstract

Large-scale protracted outbreaks can be prevented through early detection, notification, and rapid control. We assessed trends in timeliness of detecting and responding to outbreaks in the African Region reported to the World Health Organization during 2017–2019. We computed the median time to each outbreak milestone and assessed the rates of change over time using univariable and multivariable Cox proportional hazard regression analyses. We selected 296 outbreaks from 348 public reported health events and evaluated 184 for time to detection, 232 for time to notification, and 201 for time to end. Time to detection and end decreased over time, whereas time to notification increased. Multiple factors can account for these findings, including scaling up support to member states after the World Health Organization established its Health Emergencies Programme and support given to countries from donors and partners to strengthen their core capacities for meeting International Health Regulations.

The World Health Organization (WHO) African Region, encompassing 47 member states (*1*), carries one of the heaviest burdens of public health crises globally, including health emergencies due to disease outbreaks and humanitarian events that potentially pose international public health threats (*2**–**4*). More than 100 major public health events are reported annually in the region (*5*), which means these countries need to strengthen their capacities for early detection, notification, and response to mitigate their effect. These capacities are defined by the legally binding International Health Regulations 2005 (IHR 2005), which requires signatory member states “to prevent, protect against, control and provide a public health response to the international spread of disease” (*6*).

Using the response to the 2014–2016 Ebola virus disease outbreak in West Africa as a model, WHO established the WHO Health Emergencies (WHE) Programme in 2016 to help member states gain capacities to prevent, prepare for, detect, report on, respond to, and recover from public health emergencies (*7*). WHE provides technical support for member states to set up strong disease surveillance programs for detecting public health events early and to enhance capacities for responding rapidly, which has resulted in an increased number of events being detected and reported by member states (*8*). 

Evaluating the performance of outbreak detection, notification, and control activities in the WHO African Region, as well as understanding the factors associated with changes in the timeliness of response over time, could provide key insights into factors that enable or inhibit effective outbreak response, and provide guidance for identifying or refining adapted interventions to improve performance. Metrics have been proposed for objectively and quantitatively evaluating this performance by systematically capturing and analyzing data on timeliness for reaching key milestones in outbreak detection and response (*9*). 

For this study, we quantitatively assessed outbreak response in the African Region by measuring the timeliness and associated factors of reaching 3 milestones in outbreak response—detection, notification, and end—to determine if progress has been achieved since 2017 in the African Region. Our results provide a baseline metric for assessing progress towards improved disease surveillance and outbreak response in the region. 

## Methods

We undertook a retrospective study of responses to all substantiated disease outbreaks reported to WHO by member states in the African Region during 2017–2019. Outbreaks were reported using the Integrated Disease Surveillance and Response (IDSR) strategy. A substantiated outbreak was defined as one whose hazard was confirmed or in which the occurrence of human cases was clearly in excess of normal expectancy; that is, the epidemic threshold was reached or surpassed. The substantiated disease outbreaks constituted a subset of all the public health events reported to WHO, which also encompasses humanitarian and other health emergencies. 

The primary data source was the public health event database maintained by the WHE Programme at the Regional Office for Africa, which contains all formally reported public health events in the African Region including those verified through epidemic intelligence activities. Variables captured include country name, event name, etiology, date of onset, date of detection, date of notification to WHO, date of end of outbreak, and total number of cases and deaths, as well as a short description of events. Two additional variables were derived from the public health event database: each country reporting an outbreak was assigned to the Central, West, Eastern, or Southern African subregion on the basis of the WHO intercountry support team structure (*10*), and disease category was assigned on the basis of either similarity in mode of transmission, type of etiologic agent, or diseases requiring the same type of public health intervention (e.g., vaccination). 

We used secondary sources to complete missing data, including the WHO event management system, IDSR weekly bulletins produced by member states in the African Region, outbreak investigation and situation reports submitted to WHO via email, published articles in peer-reviewed scientific journals, and the World Development Indicators database. The event management system is an online central repository for all globally reported events that may constitute a public health risk to countries through the international spread of disease or that may require a coordinated international response as required by IHR 2005 regulations (*11*). The World Development Indicators database is the World Bank’s compilation of cross-country data on development, providing internationally comparable statistics on global development and the fight against poverty (*12*). We extracted data from the database for 2017–2019 for selected variables that could influence our outcomes of interest (*13*): income level, population density, health expenditure as a percentage of gross domestic product, the percentage of internally displaced population, and the refugee population living in the countries in which the outbreaks occurred. We chose these predictor variables guided by available theoretical and empirical literature to determine how well our results agree with those of other published studies concerning these predictors and also to identify what is unique to the African Region. 

Humanitarian emergencies and other public health events reported to WHO that did not constitute a disease outbreak were initially excluded because we could not estimate key milestone dates and their end dates did not necessarily depend on timely implementation of public health response given the context in which they occurred. Only substantiated disease outbreaks were selected for the study. Before analyzing the outcomes of interest, for each variable we discarded data from any outbreaks missing key milestone dates, while retaining data from that outbreak in other analyses that did not involve variables with missing data (*14*). We calculated the proportion of outbreaks sampled across each variable subcategory (e.g., by income level) and compared them with the proportion in all outbreaks to ensure representativeness and make inferences. 

We adapted the definition of milestones used by Chan et al. (*15*). We chose 4 key dates from each outbreak to determine milestones for the analysis: dates of onset, detection, notification, and end of outbreak. For date of onset we used the reported date of symptom onset for the first case found by the investigators. For date of detection we used the date on which national authorities were alerted to the outbreak. For date of notification we used the date the event was first reported to WHO by a member state. For date of end we used either the date when a country declared an outbreak to have ended, or a defined length of time over which no new cases were reported. As a proxy, we used twice the maximum incubation period from the date of recovery or death for the last case. We chose this definition to ensure that disease transmission was no longer occurring and the outbreak was over. 

We measured time to detection as the number of days between the dates of onset (first reported case) and detection, time to notification as the number of days between the dates of detection and notification, and time to end as the number of days between the dates of onset and end of the outbreak. A total of 29 events that began but had not ended during the study period were right censored from the study on December 31, 2019. 

We performed univariable and multivariable Cox proportional hazards regression analyses in 3 separate models to assess the rates of change over time to outbreak detection, notification, and end. Predictor variables were outbreak start year, WHO African subregion, income, number of refugees, percentage of internally displaced population, current health expenditure as percentage of gross domestic product, population density, and disease category. Each outbreak milestone was treated independently. 

We computed the median time and interquartile range (IQR) for the time to reach each of the milestones for all the outbreaks included in the study, then stratified results by year of outbreak start, WHO Africa subregion (Central, West, Eastern, or Southern), and disease category (food/waterborne, vaccine-preventable, vectorborne, viral hemorrhagic fever, or others). We used the World Bank’s classification of countries’ economies (*16*) to stratify results by income level (low vs. middle and high). We used median values (i.e., in-country value for each variable compared with median value for that variable for all African Region countries) to stratify results by number of refugees (low or high), percentage of internally displaced population (low or high), current health expenditure as percentage of gross domestic product (low or high), and population density (low or high). The results are indicated as hazard ratios (HR) with 95% CIs. An HR >1 indicates improvement in the time to each of the milestones, whereas an HR <1 signals a regression in the time to each milestone. R version 3.5.2 (https://www.r-project.org) was used for statistical analysis and ESRI ArcGIS Desktop 10.6.1 (https://desktop.arcgis.com) for mapping of outbreaks included in our study.

## Results 

**Figure 1:**
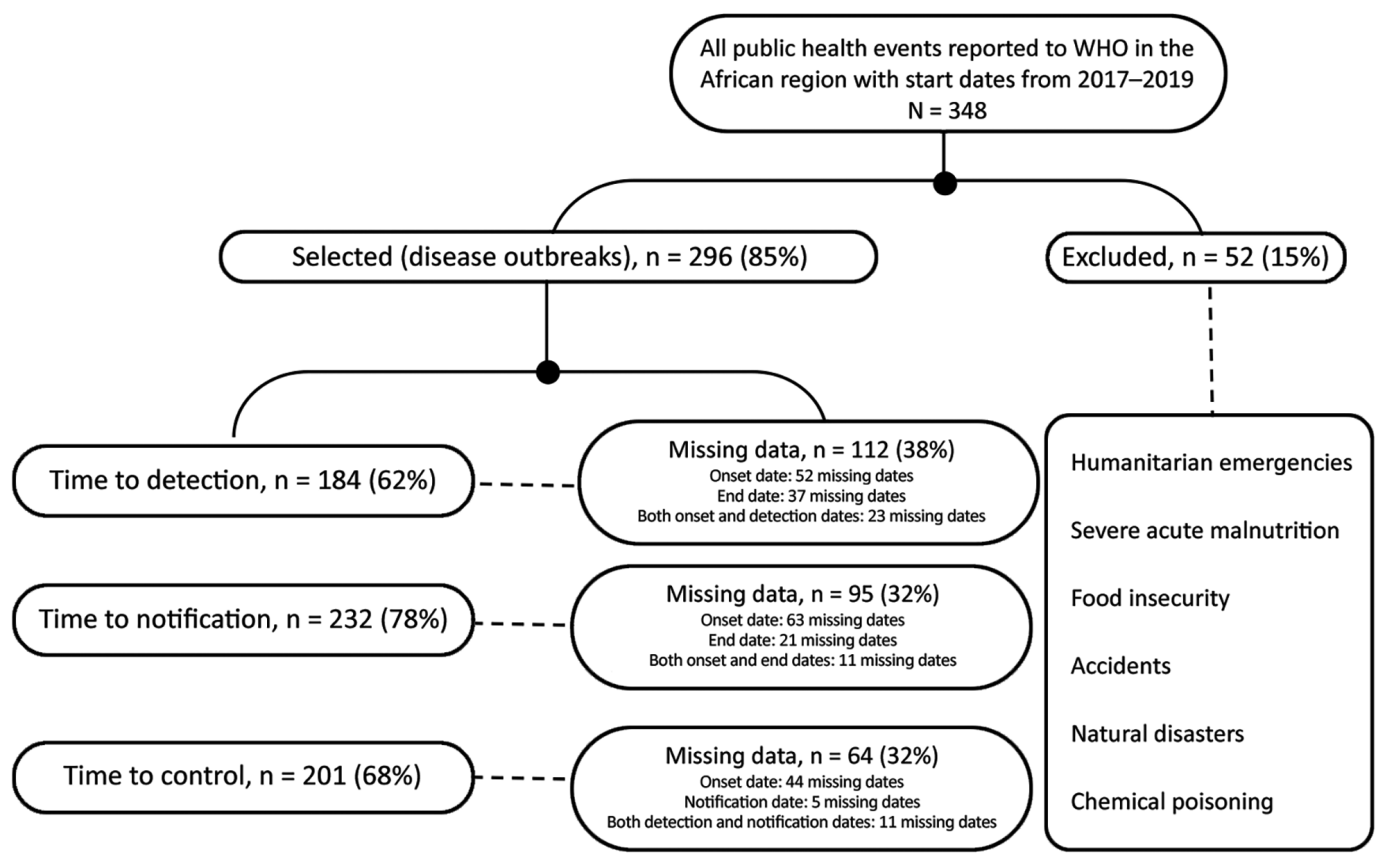
Exclusion criteria used to select subset of substantiated disease outbreaks reported to the WHO African Region, 2017–2019. WHO, World Health Organization.

Of the 348 substantiated public health events reported to WHO in the African Region from 2017 to 2019, we selected 296 disease outbreaks for the study. Key milestone dates were often missing, and therefore we only included 184 (62%) events for time to detection, 232 (78%) events for time to notification, and 201 (68%) events for time to end (Figure 1). The percentage of events included in the study for each outcome of interest slightly decreased from 2017 through 2019, after exclusion of outbreaks with missing data, across each year (Tables 1, 2). 

**Table 1:** Median time to progression for 3 outbreak milestones (detection, notification, and end) by predictor variables, WHO African Region, 2017–2019*

Categories	Total no.†	Time to detection (*15*)			Time to notification (*15*)			Time to end (*15*)	
		No. (%)‡	Median (IQR)		No. (%)‡	Median (IQR)		No. (%)‡	Median (IQR)
Income (*16*)									
Low	156	107 (68.6)	9 (2–27)		127 (81.4)	3 (0–8)		109 (53.2)	86 (37–169)
Middle and high	140	77 (55.0)	8 (2–29)		105 (75.0)	2 (0–8)		92 (44.9)	54 (24–155)
WHO subregion (*10*)									
Eastern/Southern	133	78 (58.6)	6 (1–23)		96 (72.2)	3 (0–9)		96 (46.8)	70 (30–148)
Western	102	74 (72.5)	10 (5–28)		88 (86.3)	2 (0–6)		37 (18.0)	68 (29–158)
Central	61	32 (52.5)	16 (3–33)		48 (78.7)	2 (0–9)		68 (33.2)	136 (50–221)
Outbreak start date§ (*15*)									
2017	103	75 (72.8)	14 (6–37)		87 (84.5)	1 (0–5)		62 (30.2)	131 (67–237)
2018	101	62 (61.4)	7 (1–27)		83 (82.2)	3 (0–14)		72 (35.1)	67 (25–144)
2019	87	47 (54.0)	4 (1–11)		62 (71.3)	4 (1–9)		67 (32.7)	45 (22–90)
No. refugees from elsewhere (*13*)									
Low¶	199	129 (64.8)	8 (3–28)		160 (80.4)	2 (0–8)		133 (64.9)	69 (25–166)
High¶	97	55 (56.7)	8 (1–28)		72 (74.2)	3 (0–9)		68 (33.2)	84 (43–147)
IDP, % population (*13*)									
Low¶	212	139 (65.6)	7 (2–23)		170 (80.2)	3 (0–8)		145 (70.7)	60 (25–126)
High¶	84	45 (53.6)	17 (2–37)		62 (73.8)	2 (0–9)		56 (27.3)	153 (65–227)
Disease category									
Food/waterborne	74	47 (63.5)	2 (0–7)		54 (73.0)	3 (0–6)		60 (29.3)	82 (24–154)
Vectorborne	48	24 (50.0)	7 (1–24)		34 (70.8)	3 (0–24)		31 (15.1)	138 (48–232)
Viral hemorrhagic fever	56	41 (73.2)	9 (6–17)		49 (87.5)	1 (0–4)		37 (18.0)	47 (28–82)
Vaccine-preventable	85	49 (57.6)	28 (8–50)		67 (78.8)	2 (0–15)		52 (25.4)	90 (52–175)
Other	33	23 (69.7)	11 (4–18)		28 (84.8)	5 (1–10)		21 (10.2)	42 (17–146)
Current health expenditure, % GDP (*13*)									
Low¶	162	99 (61.1)	11 (3–21)		126 (77.8)	2 (0–9)		112 (54.6)	79 (35–167)
High¶	134	85 (63.4)	7 (2–21)		106 (79.1)	3 (0–8)		89 (43.4)	77 (27–155)
Population density (*13*)									
Low¶	201	121 (60.2)	11 (3–31)		157 (78.1)	2 (0–9)		131 (63.9)	76 (36–191)
High¶	95	63 (66.3)	6 (2–18)		75 (78.9)	2 (0–5)		70 (34.1)	77 (21–130)

*GDP, gross domestic product; IDP, internally displaced persons; IQR, interquartile range. †Total number based on records of total outbreaks meeting selection criteria (total for each modality = 296).  ‡Numbers based on records of total outbreaks minus those with key missing dates (n [detection] = 184, n [notification] = 132, n [end] = 201 for each modality). Percentages are figured as no. in category/total number × 0.01. §Five dates missing in initial dataset.  ¶Low indicates < median, high indicates > median of in-country value for variable compared with that value for all countries in the African region.

**Table 2:** Number and frequency of disease outbreaks selected for the study on timeliness of outbreak milestones in the WHO African Region, 2017–2019

Outbreaks	Disease category	No. (%) outbreaks
Cholera	Food/waterborne	55 (18.6)
Measles	Vaccine-preventable	33 (11.1)
Dengue fever	Vectorborne	23 (7.8)
Crimean-Congo hemorrhagic fever	Viral hemorrhagic fever	22 (7.4)
Poliomyelitis (circulating vaccine-derived poliovirus type2)	Vaccine-preventable	17 (5.7)
Meningococcal disease	Vaccine-preventable	16 (5.4)
Lassa fever	Viral hemorrhagic fever	15 (5.1)
Anthrax	Other	13 (4.4)
Monkeypox	Other	12 (4.1)
Rift Valley fever	Viral hemorrhagic fever	12 (4.1)
Yellow fever	Vaccine-preventable	11 (3.7)
Malaria	Vectorborne	10 (3.4)
Plague	Vectorborne	6 (2.0)
Chikungunya	Vectorborne	6 (2.0)
Hepatitis E	Food/waterborne	5 (1.7)
Ebola virus disease	Viral hemorrhagic fever	5 (1.7)
Typhoid fever	Food/waterborne	4 (1.4)
Acute bloody diarrhea	Food/waterborne	4 (1.4)
Pertussis	Vaccine-preventable	3 (1.0)
Food--borne	Food/waterborne	3 (1.0)
Listeriosis	Food/waterborne	2 (0.7)
Influenza A(H1N1)	Other	2 (0.7)
Rubella	Vaccine-preventable	2 (0.7)
Aflatoxicosis	Food/waterborne	2 (0.7)
Guinea worm disease	Other	2 (0.7)
Marburg	Viral hemorrhagic fever	2 (0.7)
Leishmaniasis	Vectorborne	2 (0.7)
Botulism	Food/waterborne	1 (0.3)
Adverse effect following immunization	Other	1 (0.3)
Hepatitis A	Vaccine-preventable	1 (0.3)
Rotavirus	Vaccine-preventable	1 (0.3)
Zika virus disease	Vectorborne	1 (0.3)
Diphtheria	Vaccine-preventable	1 (0.3)
Scabies	Other	1 (0.3)
Total		296 (100.0)

**Figure 2:**
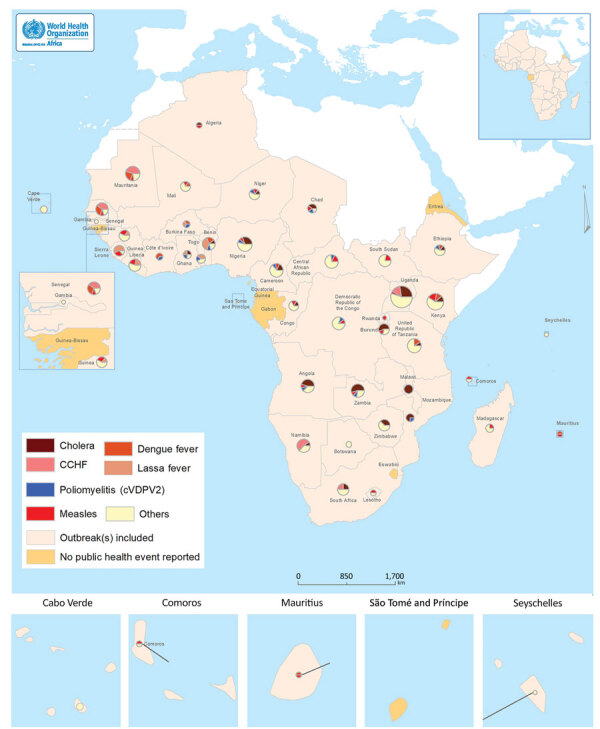
Geographic distribution of substantiated disease outbreaks selected in study of timeliness of key outbreak milestones in the WHO African Region, 2017–2019. CCHF, Crimean-Congo hemorrhagic fever; WHO, World Health Organization. cVDPV2, circulating vaccine-derived poliovirus type 2

Events meeting our selection criteria occurred in 41 out of 47 WHO member states in the African Region. Uganda registered the highest number of events (n = 21); The Gambia, Rwanda, and Seychelles registered the fewest (n = 1). São Tomé and Príncipe, Guinea-Bissau, Gabon, Equatorial Guinea, Eritrea, and Eswatini did not report any disease outbreak with a start date during the study period (Figure 2). Cholera (18.6%) was the most frequently reported outbreak disease, followed by measles (11.1%), dengue fever (7.8%), Crimean-Congo hemorrhagic fever (7.4%), poliomyelitis (5.7%), and meningococcal disease (5.4%) (Table 2).

### Overall Duration of Key Milestones 

We found an overall median time of 8 (IQR 2–28) days for time to detection, 3 (IQR 0–9) days for time to notification, and 77 (IQR 33–165) days for time to end. When analyzed by disease category, vaccine-preventable diseases had the longest median time to detection of 28 (IQR 8–50) days, whereas food/waterborne diseases had the shortest at 2 (IQR 0–7) days. The longest median time to end was 138 (IQR 48–232) days for vectorborne diseases and the shortest was 42 (IQR 17–146) days for diseases in the others category. Median times for these and the other stratifying variables—income, WHO African subregion, number of refugees, internally displaced population, current health expenditure, and population density—are shown in Table 1. 

### Changes in Duration of Key Milestones over Time 

Overall, the median time to end of outbreaks improved, decreasing from 131 (IQR 67–237) days in 2017 to 67 (IQR 25–144) days in 2018 and to 45 (IQR 22–90) days in 2019 (Table 1). The median time to detection decreased from 14 (IQR 6–37) days in 2017 to 7 days (IQR 1–27) days in 2018 and to 4 (IQR 1–11) days in 2019. The median time to notification increased over time from 1 (IQR 0–5) days in 2017 to 3 (IQR 0–14) days in 2018 and to 4 (IQR 1–9) days in 2019. 

The multivariable Cox proportional hazards models confirmed the improvement of the time to detection during 2017–2019, time to end from 2017 through 2018, and the increase in time to notification during 2017–2019 (Table 3). The other variable associated with time to detection and end was the disease category. HRs for time to detection decreased by 54% (HR 0.46, 95% CI 0.29–0.73) and time to end by 47% (HR 0.53, 95% CI 0.34–0.83) for vaccine-preventable diseases compared with food/waterborne diseases. 

**Table 3:** Results of multivariable Cox proportional hazard regression analysis of substantiated outbreaks by predictor variables, WHO African Region, 2017–2019*

Categories	Total no.†	Time to detection (*15*)	Time to notification (*15*)	Time to end (*15*)
Income (*16*)				
Low	156	Referent	Referent	Referent
Middle and high	140	1.01 (0.68–1.52)	1.11 (0.71–1.73)	1.07 (0.78–1.73)
Significance		p = 0.5147	p = 0.6289	p = 0.1513
WHO subregion (*10*)				
Eastern and Southern	133	Referent	Referent	Referent
Western	102	1.02 (0.52–1.46)	0.84 (0.47–1.50)	0.94 (0.56–1.54)
Central	61	0.81 (0.49–1.38)	1.13 (0.65–1.97)	0.55 (0.33–0.90)
Significance		p = 0.0427	p = 0.6784	p = 0.0608
Outbreak start date‡ (*15*)				
2017	103	Referent	Referent	Referent
2018	101	1.68 (1.16–2.34)	0.46 (0.31–0.70)	1.57 (1.08–2.27)
2019	87	2.59 (1.71–3.94)	0.40 (0.25–0.64)	0.94 (0.63–1.43)
Significance		p = 0.0000	p = 0.0000	p = 0.0182
No. refugees from elsewhere (*13*)				
Low‡	199	Referent	Referent	Referent
High‡	97	1.61 (0.96–2.70)	0.82 (0.45–1.48)	0.80 (0.48–1.33)
Significance		p = 0.2017	p = 0.8066	p = 0.0236
IDP, % population (*13*)				
Low‡	212	Referent	Referent	Referent
High‡	84	1.01 (0.64–2.70)	1.38 (0.85–2.23)	0.70 (0.47–1.04)
Significance		p = 0.8446	p = 0.2843	p = 0.0163
Disease category (*13*)				
Food/waterborne	74	Referent	Referent	Referent
Vectorborne	48	0.59 (0.34–1.03)	1.18 (0.63–2.22)	0.89 (0.54–1.45)
Viral hemorrhagic fever	56	0.40 (0.25–0.66)	1.57 (0.89–2.77)	1.20 (0.75–1.91)
Vaccine-preventable	85	0.46 (0.29–0.73)	1.19 (0.69–2.05)	0.53 (0.34–0.83)
Other	33	0.44 (0.34–1.05)	0.91 (0.47–1.78)	1.45 (0.85–2.46)
Significance		p = 0.0023	p = 0.4704	p = 0.0014
Current health expenditure, % GDP (*13*)				
Low§	162	Referent	Referent	Referent
High§	134	1.11 (0.75–1.65)	0.93 (0.58–1.49)	1.02 (0.69–1.51)
Significance		p = 0.5415	p = 0.7700	p = 0.9354
Population density (*13*)				
Low§	201	Referent	Referent	Referent
High§	95	1.10 (0.74–1.61)	0.98 (0.64–1.49)	0.96 (0.67–1.38)
Significance		p = 0.6555	p = 0.9258	p = 0.8251

*GDP, gross domestic product; IDP, internally displaced persons; IQR, interquartile range. †Numbers, based on records of total outbreaks meeting selection criteria (total for each modality = 296).  ‡Five dates missing in initial dataset.  §Low indicates < median, high indicates > median of in-country value for variable compared with that value for all countries in the African region.

## Discussion 

Early detection and rapid response to outbreaks greatly contributed to reducing the illness and death rates during these events. Timeliness of response is an essential element of surveillance systems (*17**,**18*). Systematically capturing and analyzing the timeliness of key milestones in outbreak detection and response can also provide critical information to public health decision makers and stakeholders on progress made towards implementing IHR requirements. Our analyses explored trends in the timeliness of detection, notification, and end of outbreaks over 3 years (2017–2019) in the WHO African Region and predictor variables that could explain changes. Overall, the findings showed a decrease in the median time to detection and end of outbreaks over the years, signaling an improvement in capacities in these areas. In contrast, the median time to notification increased. 

Our finding of overall improvement in time to detection of outbreaks in the African Region was consistent with 2 other studies, although the period studied, geography, and variables analyzed differed (*15**,**19*). In our study, the disease category was associated with differences in time to detection. Time to clinical diagnosis and laboratory confirmation of a disease affect time to detection. The median time to detection of food/waterborne disease outbreaks was found to be shorter than for other disease categories. Our final dataset contained a high number of food/waterborne diseases, with outbreaks of cholera the most frequent. Cholera has a relatively short incubation period compared with other diseases and the potential to spread rapidly, causing large-scale outbreaks (*20*). The main approach to clinical diagnosis and surveillance of cholera has been the use of the case definitions (*21*) contained in the IDSR guidelines. The scale-up of IDSR in recent years (*22*), coupled with the short incubation period, contribute to early detection of outbreaks of cholera. In addition, laboratory capacities for confirming *Vibrio cholerae,* particularly in cholera-prone settings, have improved (*23*), reducing the time needed to confirm outbreaks. However, challenges remain, as surveillance and diagnostic capacities across member states vary and serious gaps exist (*24*). 

The lower hazard ratio for time to detection of vaccine-preventable diseases compared with food/waterborne diseases could be explained in the context of the outbreak threshold for these diseases and how they are monitored. The IDSR outbreak threshold for cholera is one confirmed case while most vaccine-preventable diseases require several cases over a period of time to reach the epidemic threshold (*21*). Cholera outbreaks, therefore, are more likely to generate a quick alarm. Furthermore, monitoring of diseases reaching an outbreak threshold in the region continues to be performed mainly through indicator-based surveillance that relies on structured weekly IDSR reports from health facilities. This structure could likely result in delays in early detection of vaccine-preventable disease outbreaks because this system captures only those cases from people visiting a health facility. Event-based surveillance systems are designed to address this limitation by capturing reports from a wide variety of sources. For example, introducing community event-based surveillance contributed to increased reporting of suspected cases of measles in Liberia, leading to early detection of measles outbreaks (*25*). However, event-based surveillance is not yet well developed in most countries in the region and there remain major gaps in its implementation (*22*). 

No other predictor variable had a notable association with time to detection. Differences in national income levels and health expenditures by country GDP could provide plausible explanations for variations in the efficiency of health systems (*26*), with higher expenditures possibly leading to capacities gained for detecting outbreaks faster, even though we did not find any significant association (p>0.05) with the time to detection in this study. Also, delayed detection in the Central African subregion could be partly attributed to protracted humanitarian crises resulting from armed conflicts that have plagued many countries in this subregion (*27*,*28*). Notwithstanding, our study also did not find a significant association (p>0.05). 

Other than outbreak start year, none of the predictor variables had a statistically significant association (p>0.05) with the time to notification. Rapid notification is aimed at preventing or limiting the international spread of diseases and avoiding disruption to international trade (*29*). Under this guideline, member states need to notify WHO within 24 hours of assessment of any event in their territory “that may constitute a public health emergency of international concern” (*6*). Reversing the increase over the years in time to notification will require continuous engagement with member states and capacity building for the national IHR focal points. Initiatives such as the open-access online “Global Health Security, Solidarity, and Sustainability through the International Health Regulations” course (*30*) may help enhance this capacity. 

Many countries in the WHO African Region continue to experience recurrent disease outbreaks. Lessons gained from each outbreak response have helped to improve response to subsequent outbreaks, a possible reason for the reduction in time to end. The lower HR for controlling outbreaks of vaccine-preventable diseases compared with food/waterborne disease outbreaks may reflect challenges associated with vaccine acquisition, uptake, and access, particularly in hard-to-reach areas. 

Our list of predictor variables was not exhaustive and several other factors may have influenced the improvement in time to detection and time to end, for example, the roles of WHE and other high-level initiatives and partnerships. WHE’s enhanced use of a digital disease detection approach through media monitoring platforms such as Hazard Detection and Risk Assessment and Epidemic Intelligence from Open Sources (*31**–**33*) has expanded the window of opportunity for capturing event-based surveillance information. In 2018, about one quarter of the events in the African Region were verified by member states and reported to WHO following media monitoring activities undertaken at the Regional Office for Africa (*34*). In addition, several other high-level initiatives have been undertaken in the aftermath of the 2014–2016 Ebola outbreak to bolster global capacities for preparedness and response to infectious disease outbreaks (*35*).

There are 3 main limitations of the study. First, key milestone dates were missing in some of the reports sent to WHO, which excluded some outbreaks from the analysis. This omission may have had some effect on the findings, although efforts were made to ensure that the sample was representative of the study population of events (Appendix). Second, the number of outbreaks considered in our analysis is not an exhaustive list of all the outbreaks that occurred in the region. Only those reported to WHO by member states on the basis of IHR (2005) requirements, using the Annex 2 decision instrument (*36*), were included in the study. Third, the application of right censoring at the end of the study may have affected the results, especially for time to end of outbreaks in 2019, although the difference is unlikely to be substantial because relatively few events were right censored. 

In spite of these limitations, our study shows that outbreak metrics can be collected and measured to allow countries to monitor the timeliness of outbreak detection and response and provides an estimate of improvement of outbreak detection and end over time. The next step is to enable countries to set up systems in which these measurements are routinely collected, analyzed, and used to improve surveillance and response interventions. 

In conclusion, our study has established that the use of simple, easy-to-collect, and verifiable metrics is key to monitoring the timeliness of outbreak detection and response in the WHO African Region. The findings of improvement in early outbreak detection and rapid control over the studied period should be interpreted within the context of the variations and multiple factors that influence these outcomes. Further studies could shed better light on these variations within the member states, and across subregions and disease categories. The momentum needs to be sustained and member states supported in building capacities for early detection, notification, and rapid control of outbreaks. It is equally necessary to support member states to enable them to track key milestones systematically for continuous measurement of outbreak response performance.

Appendix Representativeness of samples from the population of outbreaks reported, WHO African Region, 2017–2019.
