# Supplementary material for: Measuring Timeliness of Outbreak Response in the World Health Organization African Region, 2017–2019
Source: Emerg Infect Dis. 2020 Nov;26(11):2555–64. doi: 10.3201/eid2611.191766 (PMC7588517; doi:10.3201/eid2611.191766)
Supplement: Appendix — Representativeness of samples from the population of outbreaks reported, WHO African Region, 2017–2019. [file 19-1766-Techapp-s1.pdf]

Article DOI: https://doi.org/10.3201/eid2610.191766

Measuring Timeliness of Outbreak Response in the World Health Organization African Region, 2017–2019

Appendix

Appendix Table. Representativeness of samples from the population of outbreaks reported, WHO African Region, 2017–2019

		Total (N = 296)		Time to detection		Time to notification		Time to control	
Variable	Modalities	N*	%	n†	%	n†	%	n†	%
Income (*16*)	Low	156	52.7	107	58.2	127	54.7	109	54.2
	Middle and high	140	47.3	77	41.8	105	45.3	92	45.8
WHO subregion (*10*)	Eastern/Southern	133	44.9	78	42.4	96	41.4	96	47.8
	Western	102	34.5	74	40.2	88	37.9	37	18.4
	Central	61	20.6	32	17.4	48	20.7	68	33.8
Outbreak start date (*15*)	2017	103	34.8‡	75	40.8	87	37.5	62	30.8
	2018	101	33.1‡	62	33.7	83	35.8	72	35.8
	2019	87	29.4‡	47	25.5	62	26.7	67	33.4
No. refugees from elsewhere (*13*)	low§	199	67.2	129	70.1	160	69.0	133	66.2
	high§	97	32.8	55	29.9	72	31.0	68	33.8
IDP (% population) (*13*)	low§	212	71.6	139	75.5	170	73.3	145	72.1
	high§	84	28.4	45	24.5	62	26.7	56	27.9
Disease category	Food-/waterborne	74	25.0	47	25.5	54	23.3	60	29.9
	Vector-borne	48	16.2	24	13.0	34	14.7	31	15.4
	Viral hemorrhagic fever	56	18.9	41	22.4	49	21.1	37	18.4
	Vaccine-preventable	85	28.7	49	26.6	67	28.9	52	25.9
	Other	33	11.2	23	12.5	28	12.0	21	10.4
Current health expenditure (% GDP) (*13*)	low§	162	54.7	99	53.8	126	54.3	112	55.7
	high§	134	45.3	85	46.2	106	45.7	89	44.3
Population density (*13*)	low§	201	67.9	121	65.8	157	67.7	131	65.2
	high§	95	32.1	63	34.2	75	32.3	70	34.8

GDP, gross domestic product; IDP, internally displaced persons; IQR, interquartile range; Med., median
*N, based on records of total outbreaks meeting selection criteria (total for each modality = 296)
†n, based on records of total outbreaks minus those with key missing dates (n = 184 [detection], n = 132 [notification], n = 201 [control] for each modality)
‡Because 5 reports in the initial dataset were missing key dates, the percentages do not total 100%. Data from those reports were not included in the findings.
§Low indicates ≤ median, high indicates > median of in-country value for variable compared with that value for all countries in the African region
